# Gilbert’s Syndrome With Diabetes Mellitus

**DOI:** 10.7759/cureus.16557

**Published:** 2021-07-22

**Authors:** LIU HAIXIA, Ruiman Li

**Affiliations:** 1 Obstetrics and Gynecology, The First Affiliated Hospital of Jinan University, Guangzhou, CHN

**Keywords:** gilbert’s syndrome, diabetes mellitus, pregnancy, hyperbilirubinemia, gdm

## Abstract

Gilbert's syndrome (GS) could lead to the high bilirubin, and gestational diabetes mellitus might reverse this index. A primigravida with a pregnancy of 38 weeks and 4 days was identified having gestational diabetes mellitus (GDM) with Gilbert's syndrome. General laboratory tests were normal except mild unconjugated hyperbilirubinaemia and hyperglycemia. The delivery process was going well with completely delivered placenta and fetal membrane, and I° turbid amniotic fluid. The newborn was noted to have high bilirubin level which reversed after a few days of treatment. Gilbert's syndrome is rare in obstetric practice with the virtually decreased activity of uridine diphosphate glucuronosyl transferase (UDPGT). This is the first report to confirm that pregnant women with Gilbert's syndrome and gestational diabetes could give birth normally without significant adverse symptoms, except for jaundice.

## Introduction

Gilbert's syndrome is a common autosomal dominant hereditary condition with incomplete penetrance and characterized by intermittent unconjugated hyperbilirubinemia in the absence of hepatocellular disease or hemolysis. Gestational diabetes mellitus (GDM) refers to diagnosis of diabetes at 24 to 28 weeks of gestation. Diagnosis of diabetes in early pregnancy is more consistent with previously undiagnosed type 2 diabetes.

## Case presentation

A 28-year-old women with a pregnancy of 38 weeks and 2 days was admitted to the First Affiliated Hospital of Jinan University with amenorrhea. The patient had stable vital signs, and mild yellow skin discoloration was noted. Scan showed a single live fetus corresponding to dates with adequate amniotic fluid. The oral glucose tolerance test (OGTT) results at pregnancy of 24 weeks was 5.76mmol/L, 1.37mmol/L, and 9.39mmol/L corresponding to fasting blood sugar (FBS), 1-hour postprandial blood glucose (1hPG) and 2-hour postprandial blood glucose (2hPG). Diagnostic criteria of 75g OGTT: fasting blood glucose, 1 hour and 2 hours after taking glucose were <5.1 mmol/L, <10.0 mmol/L and <8.5 mmol/L, respectively. GDM is diagnosed when the blood glucose value at any point reaches or exceeds the above criteria. The concentration of bilirubin were 137.6-221.3umol/L, including the direct bilirubin and indirect bilirubin. The investigations showed that her urobilinogen concentration was at 34-50umol/L. Complete blood count (CBC) in other hospital showed that her Hb was 100g/L, glycosylated hemogobin was 4.2%, and fasting plasma glucose (FPG) was 4.11mmol/L, respectively. Liver Function Tests (LFT) results showed that her total bilirubin was 189.9umol/L (Table 1), direct bilirubin at 12.9umol/L, indirect bilirubin at 177 umol/L, total bile acid (TBA) at 2.8umol/L, alanine aminotransferase (AST) at 8U/L and alanine transaminase (ALT) at 5.4U/L,respectively. No abnormalities were found in the results detected by hepatic-bladder-pancreatic-splenic-ultrasonography. The admission diagnosis results shown that patient had Gilbert’s syndrome coupled with GDM (White A1) and mild anemia (100g/L).

**Table 1:** Pre-pregnancy liver function ALT, Alanine transaminase; AST, Alanine aminotransferase;

Date	Total bilirubin( umol/L )	Conjugated bilirubin( umol/L )	Unconjugated bilirubin( umol/L )	ALT( U/L )	AST( U/L )
2019.07.15	89.9	12.9	177	5.4	8
2019.08.22	194.3	12.1	182.2	6	10
2019.09.20	227.5	9.1	218.4	12	19

By obtaining a thorough history with the aim of questioning the patient for Gilbert’s syndrome, we found that patient showed no signs of jaundice. She was found to present whole skin and mucosal jaundice 1 week after birth, revealing neonatal jaundice. Unfortunately, the jaundice was not reversed after phototherapy treatment in local hospital. Subsequently, the patient was treated with traditional Chinese medicine and acupuncture. Despite this, symptoms were not ameliorated, and diagnosis was undetermined. Liver puncture results detected in Beijing at her adult age showed that no abnormalities were found in her hemopoietic system, liver function, liver structure and hepatobiliary systems, in addition to Gilbert syndrome. Whenever she got fever, her hemobilirubin was dramatically elevated to 400umol/L. Notably, her skin and mucosal jaundice was mildly relieved when accessed to sunshine or being happy. Patient's physique was tiny, but she did not frequently have fever since she did exercise in middle school.

Patient deliverd a live female infant by spontaneous vaginal delivery at 38 weeks and 4 days of gestation, and her amniotic fluid was turbid at I°. The infant was sent to neonatal department due to elevated bilirubin and discharged with the recovered normal bilirubin concentration after treatment. The repeat maternal CBC and liver function tests, shown that her total bilirubin was 243.6umol/L, direct bilirubin 12.2umol/L, indirect bilirubin 231.4umol/L, TBA 6.3umol/L, ASL 8U/L and ALT 5.4U/L, respectively (Table 2). The follow-up investigation was completed at 42 days after the delivery, and the patient returned to normal status.

**Table 2:** Postpartum iver function ALT, Alanine transaminase; AST, Alanine aminotransferase;

Date	Total bilirubin(umol/L)	Conjugated bilirubin(umol/L)	Unconjugated bilirubin(umol/L)	ALT（U/L）	AST（U/L）
2019.09.24	243.6	12.2	231.8	8	5.4

## Discussion

Gilbert syndrome

Gilbert syndrome is a common autosomal dominant hereditary condition with incomplete penetrance, which is characterized by intermittent unconjugated hyperbilirubinemia in the absence of hepatocellular disease or hemolysis [1]. In patients with Gilbert’s syndrome, uridine diphosphate-glucuronyl transferase activity was reduced to 30% of the normal, resulting in indirect hyperbilirubinemia. In its typical form, hyperbilirubinemia was firstly noticed as intermittent mild jaundice in adolescence. However, Gilbert’s syndrome combined with other prevailing conditions such as breast feeding, G-6-PD deficiency, thalassemia, spherocytosis or cystic fibrosis might potentiate severe hyperbilirubinemia and/or cholelithiasis. In a study by Kamal et al. [2], logistic regression showed that pregnancy was a significant risk factor for clinical jaundice episodes. The study demonstrated that pregnancy represented an important risk factor for exacerbation of indirect hyperbilirubinemia starting from the first trimester and persisting throughout pregnancy and part of the post-partum period. In a study by Mohan et al., the primigravida presented at 32 weeks of gestation with vomiting, myalgia and jaundice. In a study by Yang et al., the primigravida presented at 28 weeks of gestation with body itching and swollen gums; she had bleeding gums while brushing her teeth. However, our case is different from other reported cases as, in addition to jaundice, the course of pregnancy patients has no other adverse symptoms such as no signs of itching and abdominal pain. According to our patient, when she was exposed to sunshine or when she was happy, her skin and mucosal jaundice were mildly relieved, and it was reversed to normal levels at 42 days after the delivery.

Gestational diabetes mellitus

Gestational diabetes covers individuals with pre-pregnancy diabetes mellitus and gestational diabetes mellitus. More than 90% of pregnant women with diabetes were having GDM, and the incidence of GDM increased significantly with the changes of diagnostic criteria [5]. The trial results in this case showed that OGTT at 24 gestation weeks were 5.76 (FBS), 11.37 (1hPG) and 9.39mmol/L (2hPG), indicating gestational diabetes mellitus (WhiteA1). We can use scientific methods such as education, diet and exercise to control blood sugar, closely monitor blood sugar in patients, and significantly improve pregnancy outcomes for pregnant women and fetuses. Along with the increased incidence of respiratory distress syndrome, neonatal hypoglycemia, hypocalcemia and hypomagnesemia, the occurrences of hyperbilirubinemia and polycythemia were higher than that in normal pregnant women. Unconjugated bilirubin levels were usually detected at 10-16 weeks of gestation and remained high levels throughout pregnancy or some postpartum periods. In this case, neonatal bilirubin was significantly increased after birth. This might be caused by maternal gestational diabetes, rather than Gilbert's syndrome due to the genetic factors of maternal bilirubin.

The role of hyperbilirubinemia and diabetes mellitus

The underlying cause as to why our case differs from other reports may lie in the interrelationship between the two diseases, hyperbilirubinemia and diabetes mellitus, discussed in the relevant research. Bilirubin was negatively associated with T2DM[6]. The increase of total bilirubin in serum may be related to the decrease of diabetes prevalence by using the database from the US National Health and Nutrition Examination Survey[7]. High bilirubin is usually associated with a lower risk of diabetes. However, bilirubin can affect the oxidative effects and lowering of lipids within cells [8]. In addition to a radical scavenging activity, the important reason for the increase of superoxide production in diabetic vascular tissue may be related to the inhibition effect of bilirubin on the activity of NAD(P)H oxidase, so that sustained hyperbilirubinemia inhibits oxidative stress and prevents the development of vascular complications [9].

## Conclusions

This paper emphasizes the relationship between hyperbilirubinemia and diabetes mellitus. Our patient did not have the same severe symptoms or pregnant outcomes as other reported patients. The confirmation of diagnosis ensured appropriate treatment and avoided over-treatment. After delivery, all symptoms and examination indexes returned to normal. If a pregnant woman is diagnosed with Gilbert syndrome, first, we should rule out other diseases, pay attention to nausea and vomiting, skin and sclera yellow dye, systemic itching and other manifestations. This case gave the meaningful information for further treatment or diagnosis of pregnancy having Gilbert’s syndrome coupled with diabetes mellitus.
